# Revision surgery for chronically discharging mastoid cavities: mastoid obliteration with canal wall reconstruction versus non-obliteration surgery

**DOI:** 10.1007/s00405-021-07138-0

**Published:** 2021-10-27

**Authors:** Hylke F. E. van der Toom, Marc P. van der Schroeff, Tim L. Molenaar, Mick Metselaar, Anne van Linge, Jantien L. Vroegop, Robert J. Pauw

**Affiliations:** Department of Otorhinolaryngology and Head and Neck Surgery, Erasmus Medical Center, Doctor Molewaterplein 40, 3015 GD Rotterdam, The Netherlands

**Keywords:** Mastoid cavity, Mastoid bowl, Revision, Obliteration, Otitis, Cholesteatoma

## Abstract

**Purpose:**

To evaluate the surgical results of revision canal wall down (CWD) surgery for chronically discharging mastoid cavities and to compare the non-obliteration approach to mastoid obliteration with canal wall reconstruction.

**Methods:**

This is a retrospective cohort study. All adult patients (≥ 18 years) who underwent revision surgery for chronically draining mastoid cavities between January 2013 and January 2020 were included. Primary outcome measures included the dry ear rate, complications and postoperative hearing.

**Results:**

79 ears were included; 56 ears received revision CWD with mastoid obliteration and posterior canal wall reconstruction and 23 ears received CWD without mastoid obliteration. The dry ear rate at the most recent outpatient clinic visit (median 28.0 months postoperative) was significantly higher in the obliteration group with 96.4% compared to 73.9% for the non-obliteration group (*p* = .002). There were no differences in audiological outcome and incidence of complications between the two techniques.

**Conclusion:**

We show that in our study population revision CWD surgery with mastoid obliteration and posterior canal wall reconstruction is superior to revision CWD surgery without mastoid obliteration in the management of chronically discharging mastoid cavities. In the obliteration group, a dry ear was achieved in 96.4% as this was 73.9% in the non-obliteration group. We found no differences in audiological outcome and in incidence of complications between the two techniques.

**Supplementary Information:**

The online version contains supplementary material available at 10.1007/s00405-021-07138-0.

## Introduction

While obliteration techniques in the surgical management of cholesteatoma gain popularity [1], cholesteatoma surgery is still often performed using the traditional canal wall up (CWU) or canal wall down (CWD) approach without obliteration. Although the recurrent and residual cholesteatoma rates after CWD without obliteration are lower compared to CWU without obliteration [2, 3], the CWD technique carries several disadvantages caused by the loss of self-cleaning capacity of the ear such as chronical discharge and infection of the persistent mastoid bowl, need for regular maintenance cleaning at the outpatient clinic, caloric-induced dizziness after temperature or pressure changes, the need for precautionary water avoidance measures and difficulty to fit hearing aids [4–8]. Reasons for surgical failure after CWD surgery include residual infected mastoid air cells, large mastoid cavities, high facial ridge, bony overhang in the mastoid cavity, tympanic membrane perforations, narrow meatus and cholesteatoma [9–11]. Discharging mastoid bowls are usually successfully controlled by frequent maintenance cleaning and topical therapy. However, in a small portion of patients, intensive topical treatment does not lead to disease control. In such cases, surgical revision may be necessary to achieve a stable ear. Several surgical techniques have been described in the management of the chronically discharging mastoid cavity, including revision canal wall down mastoidectomy with or without mastoid obliteration and with or without reconstruction of the posterior canal wall [4, 8–17]. The main goal of these interventions is to achieve the highest possible postoperative long-term dry ear rate. While the aforementioned studies have reported results using either obliteration or non-obliteration techniques alone, there is to our knowledge no study that compares mastoid obliteration with canal wall reconstruction to non-obliteration surgery in the management of chronically discharging mastoid cavities. Therefore, it would be interesting to compare the two surgical techniques with regards to the postoperative dry ear rate, complications and postoperative hearing.

In our tertiary referral center, obliteration of the mastoid with posterior canal wall reconstruction for the management of chronically discharging mastoid bowls was introduced in 2013 and gradually replaced the revision canal wall down mastoidectomy without obliteration and canal wall reconstruction. As these different approaches were performed by the same otologic surgeons, this cohort lends well for comparison of the surgical outcome of the two techniques. Therefore, the aim of this study is to evaluate the surgical results of revision surgery for chronically discharging mastoid cavities and to compare the non-obliteration approach to mastoid obliteration with posterior canal wall reconstruction.

## Materials and methods

### Patients

A retrospective cohort study was performed at our department with approval of the medical research ethical committee. All adult patients (≥ 18 years) who underwent revision surgery for chronically draining mastoid cavities between January 2013 and January 2020 were included. All patients were previously adequately but unsuccessfully treated with topical therapy. The following variables were retrieved from electronic patient records: patient characteristics, indication for surgery (chronically infection, recurrent or residual cholesteatoma, bothersome maintenance cleaning), pre-operative symptoms (discharge, pain, vertigo), tympanic membrane status, date of surgery, surgeon, whether or not mastoid obliteration with posterior canal wall reconstruction was performed, reasons for previous surgical failure (high facial ridge, residual mastoid cells, bony overhang, cholesteatoma, tympanic membrane perforation, narrow meatus), administration of antibiotics perioperatively, ossicular chain status (intact chain, absent incus, absent stapes, fixated footplate), ossicular chain reconstruction (no reconstruction due to intact chain, no reconstruction but damaged chain, incus interposition, partial ossicular replacement prosthesis (PORP), total ossicular replacement prosthesis (TORP), tympanic membrane to stapes (type III reconstruction), tympanic membrane directly to footplate), pre- and postoperative hearing (air conduction (AC), bone conduction (BC) and air–bone gap (ABG) for 0.5, 1, 2 and 4 kHz), post-operative symptoms (discharge, pain, vertigo), postoperative frequency of maintenance cleaning at outpatient clinic, postoperative otoscopy at the outpatient clinic at different timepoints. The postoperative otoscopy was scored as 1) fully dry, 2) minimal discharge or 3) infection.

### Surgical technique

The surgical technique for mastoid obliteration with posterior canal wall reconstruction was performed as described by Vercruysse et al [8]. Surgery was performed under general anesthesia. In case of obliteration, two grams of cefazolin was administrated intravenously prior to surgery and eventually repeated after 4 h. If necessary, a M-meatoplasty [18] was performed. After a wide retro-auricular question mark-shaped incision, the mastoid bowl was exposed and granulation tissue was removed. Remaining mastoid cells were removed and in case of obliteration and posterior canal wall reconstruction, cortical bone dust and cortical bone chips or conchal cartilage were collected and stored in a rifamycin solution (Sanofi-Aventis U.S. LLC, Bridgewater, New Jersey, USA. Rifadin® 600 mg powder for infusion with 10 mL solvent for solution and 20 mL 0.9% sodium chloride). After removal of all accessible cell tracts, possible bony overhang, possible cholesteatoma and/or after lowering the facial ridge, in case of mastoid obliteration, the posterior canal wall was reconstructed with cartilage or bone chips and covered with temporal fascia or a mid-temporal artery flap followed by mastoid obliteration with bone dust upon the level of the mastoid cortex. When no sufficient autologous bone dust was available for obliteration, bioactive glass granules (Bonealive^®^, Bonalive Biomaterials Ltd., Turku, Finland) were used. In most cases when no obliteration was performed, the mastoid was smoothened with some bone dust. In these cases, a mastoid cavity persisted. If possible an ossicular chain reconstruction was performed and if necessary the tympanic membrane was reconstructed with cartilage or temporal fascia. The ear canal was packed with a gauze with hydrocortisone/oxytetracycline/polymyxin B for at least 1 week and was usually re-packed after 1 week.

### Statistical analyses

Statistical analyses were performed using IBM SPSS Statistics 26 (SPSS Inc., Chicago, IL, USA) and Graphpad Prism 7.0 (GraphPad Software, La Jolla California, USA). The Chi-square test of independence was performed to examine for differences in surgical outcome between the surgical techniques. For hearing outcome and data on time intervals for postoperative maintenance cleaning (not normally distributed), the Mann–Whitney *U* test was used to assess differences between the surgical techniques. *P*-values < 0.05 were considered statistically significant.

## Results

### Patients

Between January 2013 and January 2020, 79 ears of 76 patients received revision surgery due to a chronically draining mastoid cavity. The median follow-up time was 28 months (inter quartile range (IQR) 17–55 months). Pre-operatively, 78 patients (98.7%) suffered from chronic discharge, 11 patients (13.9%) from vertigo, 6 patients (7.6%) from pain and 1 patient (1.3%) from bothersome recurrent cleaning. Obliteration of the mastoid with posterior canal wall reconstruction was performed in 56 cases (70.9%) whilst no obliteration was performed in 23 cases (29.1%). The most frequently used obliteration material was autologous bone dust (80.4%), followed by autologous bone dust together with bioactive glass granules (16.1%) and bioactive glass granules alone (3.6%). The posterior canal wall was reconstructed with bone chips in 71.4%, cartilage in 12.5% and bone chips with cartilage in 16.1%. Patient characteristics are shown in supplemental Table 1.

**Table 1:** Reasons for previous surgical failure

Reason	*N* (%)
Remaining mastoid air cells with infected mucosa	73 (92.4%)
High facial ridge	37 (46.8%)
Narrow meatus	27 (34.2%)
Remaining bony overhang	25 (31.6%)
Cholesteatoma	14 (17.7%)
Tympanic membrane perforation	13 (16.5%)
Exposed bone in mastoid bowl	3 (3.8%)

### Reasons for previous surgical failure

The reasons for previous surgical failure are shown in Table 1. Remaining mastoid air cells with infected mucosa contributed to the chronic discharge in 92.4% of cases, a high facial ridge in 46.8% of cases, a narrow meatus in 34.2% of cases, a remaining bony overhang in 31.6% of cases, cholesteatoma in 17.7% of cases, a tympanic membrane perforation in 16.5% of cases and exposed bone in the mastoid bowl in 3.8% of cases.

### Surgical outcome

The surgical outcome per technique is shown in Table 2. The dry ear rate at 8 weeks postoperatively, 1 year postoperatively and at the most recent outpatient clinic visit (median 28.0 months postoperative) was 57.1, 91.1 and 96.4%, respectively for the obliteration group and 60.9, 65.2 and 73.9%, respectively for the non-obliteration group. At 1 year postoperatively and at the most recent examination, the dry ear rate was significantly higher in the obliteration group compared to the non-obliteration group (*p* = 0.007 and *p* = 0.002, respectively). At most recent follow-up, vertigo and pain was not present in any cases in the obliteration and non-obliteration group. There was persistent need for regular maintenance cleaning in 82.1% of patients with mastoid obliteration and 100% in the non-obliteration group (*p* = 0.039) and the median time interval for maintenance cleaning at the outpatient clinic was significantly longer in the obliteration group compared to the non-obliteration group (12.0 months versus 6.0 months, *p* = 0.005).

**Table 2:** Surgical outcome per technique

		Obliteration with canal wall reconstruction	No obliteration	*P*
Number of cases		56	23	
Dry ear 8 weeks postoperatively	Yes	32 (57.1%)	9 (39.1%)	0.145
	No	24 (42.9%)	14 (60.9%)	
Dry ear 1 year postoperatively	Yes	51 (91.1%)	15 (65.2%)	**0.007**
	No	5 (8.9%)	8 (34.8%)	
Dry ear at last visit	Yes	54 (96.4%)	17 (73.9%)	**0.001**
	No	2 (3.6%)	6 (26.1%)	
Need for regular maintenance cleaning	Yes	46 (82.1%)	23 (100%)	**0.039**
	No	9 (16.1%)	0 (0%)	
Need for revision surgery	Yes	8 (14.3%)	4 (17.4%)	0.417
	No	48 (85.7%)	19 (82.6%)	
Median interval for maintenance cleaning in months (IQR)		12.0 (6.0–12.0)	6.0 (4.0–10.5)	**0.005**

Bold values indicate a significant difference (*P*-value < 0.05) between the obliteration and non-obliteration group

### Revision surgery and cholesteatoma recurrence

To achieve a dry ear, revision surgery was needed in 8 cases (14.3%) in the obliteration group and in 4 cases (17.4%) in the non-obliteration group (Table 3), mostly because of recurrent or residual cholesteatoma (3 cases, 25.0% of revisions), exposed bone (2 cases, 16.7% of revisions) or a narrow meatus (2 cases, 16.7% of revisions). In 1 case after revision CWD without obliteration, a subtotal petrosectomy was performed because of persistent discharge and fear for maintenance cleaning.

**Table 3:** Reasons for revision surgery

Reasons	*N* (%)
Revision CWD due to cholesteatoma	3 (25%)
Revision CWD due to exposed bone	2 (16.7%)
Meatoplasty	2 (16.7%)
Remaining mastoid air cells with infected mucosa	1 (8.3%)
Revision ossicular chain	1 (8.3%)
Excision subcutaneous cholesteatoma local anesthesia	1 (8.3%)
Subtotal petrosectomy	1 (8.3%)
Liquorrhea from posterior cranial fossa	1 (8.3)
Total	12

*CWD* canal wall down

During follow-up of the 14 cholesteatoma cases, a residual cholesteatoma was detected in 1 case (subcutaneously at incision site, detected at 76 months postoperatively) and a recurrent cholesteatoma in 2 cases (detected after 31 and 32 months postoperatively, respectively), resulting in a residual cholesteatoma rate of 7.1% and a recurrent cholesteatoma rate of 14.3%.

### Postoperative hearing

As shown in supplemental Table 2, there were no significant differences in perioperative ossicular chain status between the two groups. For ossicular chain reconstruction, there were no significant differences between the two groups besides for type III ossicular chain reconstruction which was more often performed in the non-obliteration group compared to the obliteration group (*p* = 0.022).

Postoperative hearing tests were available for 52 patients (92.8%) in the obliteration group and 21 (91.3%) patients in the non-obliteration group (Table 4). There were no significant differences in pre-, postoperative and change in AC threshold, BC threshold and ABG between the two groups. The median postoperative AC threshold was 46.3 dB HL in the obliteration group and 42.5 dB HL in the non-obliteration group. There were no cases of iatrogenic sensorineural hearing loss.

**Table 4:** Hearing outcome per surgical technique

	Air conduction						Bone conduction						Air–bone gap					
	Preoperative median, dB HL (IQR)	*P*	Postoperative median, dB HL(IQR)	*P*	Change, dB (IQR)	*P*	Preoperative median, dB HL(IQR)	*P*	Postoperative median, dB HL (IQR)	*P*	Change, dB (IQR)	*P*	Preoperative median, dB (IQR)	*P*	Postoperative median, dB (IQR)	*P*	Change, dB (IQR)	*P*
Obliteration with canal wall reconstruction	48.8 (38.1–64.1)	0.996	46.3 (32.5–58.4)	0.442	2.5 (–4.7–10.0)	0.262	21.9 (10.9–28.8)	0.597	20.0 (12.8–29.7)	0.306	1.3 (– 3.8–5.0)	0.406	28.8 (21.3–38.8)	0.462	25.6 (17.8–34.4)	0.530	3.8 (– 3.4–10.9)	0.164
No obliteration	46.3 (36.3–67.5)		42.5 (36.9–70.6)		0.0 (– 10.6–4.4)		21.3 (12.5–36.3)		25.0 (15.0–34.4)		0.0 (– 4.4–2.5)		25.0 (17.5–36.3)		27.5 (18.8–36.3)		2.5 (– 8.8–5.0)	

dB HL, decibel hearing level; preop, preoperative; postop, postoperative; IQR, inter-quartile range

### Complications

A postoperative otitis externa within 3 months post-surgery was observed in 3 cases (5.4%) in the obliteration group and in 4 cases (17.4%) in the non-obliteration group. Six infections were successfully controlled with topical therapy as in 1 case oral antibiotics were administered. In 2 cases (3.6%) in the obliteration group, liquorrhea occurred due to an iatrogenic defect in the cranial fossa. In 1 case, this was successfully closed during the initial procedure and in 1 case revision surgery was needed to close a defect in the posterior cranial fossa.

### Literature overview

The current literature on revision mastoidectomy for troublesome cavities is relatively scarce, as shown in a literature overview in Table 5. The dry ear rates after revision CWD range from 6–98% for the non-obliteration techniques and 40–100% for the obliteration techniques. However, it is hard to compare the different studies due to differences in sample size, follow-up duration, surgical technique and obliteration material and if whether or not a posterior canal wall reconstruction was performed.

**Table 5:** Literature overview

Source	Year	Design	No. of cases	Surgical indication	Surgical technique	Posterior canal wall reconstruction	Obliteration material	Mean follow-up period	Dry ear rate	Comments
Mills et al. [15]	1988	Retrospective	54	Chronically discharge	Revision CWD without obliteration	NA	NA	NA	59%	Cavity revision with meatoplasty resulted in a 83% dry ear rate
Black et al. [12]	1998	Retrospective	465	Retrospective	Revision CWD with and without obliteration and ablation	Proplast, cartilage, bone chips, mid-temporal flap	Palva flaps, bone pate, hydroxyapatite granules,	NA	Obliteration: 40–84% Reconstruction: 6–90% Ablation: 100%	Obliteration: 55 cases Reconstructions: 372 cases Ablation: 38 cases
Singh et al. [17]	2007	Retrospective	51	Chronically discharge, cholesteatoma	Revision CWD with obliteration	No	Mid temporal flap and musculoperiosteal flap	31 months	94%	
Phelan et al. [11]	2008	Retrospective	37	Chronically discharge, cholesteatoma, vertigo, otalgia	Revision CWD without obliteration	No	NA	36 months	98%	
Yung et al. [9]	2010	Retrospective	140	Chronically discharge, water intolerance, building up of cerumen, dizziness, cholesteatoma	Majority revision CWD with obliteration	No	Majority with hydroxyapatite granules, midtemporal flap, periosteal flap	82% had a 5-year follow-up	98%	12.1% needed surgical revision 95% water-safe
Kasenomm et al. [10]	2013	Retrospective	50	Chronically discharge, cholesteatoma	Revision CWD with and without obliteration	Conchal cartilage in some cases	Bone pate, hydroxyapatite granules, musculoperiosteal flap or no obliteration	12 months	72%	
Liu et al. [14]	2015	Retrospective	27	Chronically discharge, vertigo, cholesteatoma, mastoiditis	Revision CWD with obliteration	Free bone-connective tissue composite graft	Bone chips and bone pate	88 months	NA	85.2% water-safe 85.2% well aerated middle ear
Vercruysse et al. [8]	2016	Retrospective	50	Troublesome cavities	Revision CWD with obliteration	Cortical bone chips	Bone pate	101.8 months	94%	
Geerse et al. [13]	2017	Retrospective	122	Chronically discharge, cholesteatoma	Revision CWD with obliteration	Tragal or conchal cartilage and mid-temporal flap	Hydroxyapatite granules, sometimes with bone pate	44 months	93%	12% needed surgical revision
Uluyol et al. [4]	2017	Prospective	11	Cavity problems (accumulation of debris, vertigo)	Revision CWD with partial obliteration	Conchal cartilage	Temporalis muscle flap	6 months	100%	Comparison with 11 patients with previous CWD without reconstruction. Normalized caloric response and improvement of QOL after cavity reconstruction
Patil et al. [16]	2021	Prospective	228	Chronically discharge, cholesteatoma	Revision CWD or secondary CWD with obliteration	No	Cartilage, hydroxyapatite granules, bioactive glass granules, mid temporal flap, periosteal flap	0–186 months, 50% of cases had > 5y FU	93.7%	10% needed surgical revision 87.2% water-safe
Present study	2021	Retrospective	79	Chronically discharge, cholesteatoma	Revision CWD with and without obliteration	Cortical bone chips	Bone pate	28 months	96.4% in obliteration group 73.9% in non-obliteration group	

## Discussion

In this study, we retrospectively compared revision canal wall down surgery with mastoid obliteration and posterior canal wall reconstruction to revision canal wall down surgery without obliteration in the management of chronically discharging mastoid cavities. We show that in our study population, the obliteration technique is superior to the non-obliteration technique with regards to the dry ear rate, need for regular maintenance cleaning and interval between maintenance cleaning sessions. We found no differences in audiological outcome and in incidence of complications between the two techniques.

The incidence of discharging mastoid cavities after canal wall down surgery is estimated at 10–30% [15]. Whilst usually controllable by topical therapy and frequent maintenance cleaning, a therapy-resistant discharging mastoid cavity can be of great burden to the patient and in such cases revision surgery may be indicated. Goals of revision surgery are to create a dry, safe ear with preservation of hearing and with the lowest possible frequency of necessary outpatient clinic visits after healing of the ear. In the present study, the dry ear rate at 1-year postoperatively was 91.1% in the obliteration group and 65.2% in the non-obliteration group; these percentages were 96.4 and 73.9%, respectively at most recent follow-up (median 28.0 months postoperative). The superior results of the obliteration technique compared to the non-obliteration approach may be explained by the fact that the normal anatomy of the external ear canal is restored after obliteration while in a persistent mastoid bowl, cerumen and debris can still accumulate and potentially cause infection. While 82.1% of patients in the obliteration group still received regular maintenance cleaning at the outpatient clinic postoperatively, the median interval for maintenance cleaning was 12 months in the obliteration group, significantly longer compared to the median 6 months in the non-obliteration group.

As previous studies reported outcome on either the obliteration [4, 8–10, 12–14, 16, 17] or the non-obliteration [10–12, 15] technique alone, it remained unclear whether the outcome of these studies was a result of the surgical technique itself or a result of the surgeons’ skills. As in the present study, the two surgical techniques were performed by the same surgeons in the same institute, we show for the first time that it may actually be the obliteration technique that is robust and an important determinant factor for surgical outcome. In literature (Table 5), dry ear rates varying from 6 to 100% are reported. Due to differences in surgical technique, obliteration material (bone dust, hydroxyapatite granules, bioactive glass granules, temporal muscle flaps, Palva flaps and mid-temporal artery flaps) and follow-up period it is difficult if not impossible to compare the different studies. However, the studies in which a mastoid obliteration is performed tend to show higher postoperative dry ear rates compared to the non-obliteration studies. It is not possible to investigate the role of the posterior canal wall reconstruction on the dry ear rate in the present literature, and our study design does also not allow us to assess its role on the dry ear rate. However, we believe that a firm posterior canal wall reconstruction prevents the bone pate from dissolving and thus is an important pillar in the surgical management of the chronically discharging mastoid cavity.

We identified the reasons for previous surgical failure to be remaining mastoid air cells with infected mucosa, a high facial ridge, a narrow meatus, remaining bony overhang, cholesteatoma, a tympanic membrane perforation or exposed bone in the mastoid bowl. These findings confirm previous research in which the same factors were identified [9–11] and any otologist performing canal wall down surgery should be aware of the importance in addressing these factors to prevent surgical failure. The postoperative complications consisted mainly of infection of the external ear canal or mastoid bowl, which was observed in 3 cases (5.4%) in the obliteration group and in 4 cases (17.4%) in the non-obliteration group. However, these were mild infections which were successfully treated with topical antibiotics in 6 cases and with oral antibiotics in 1 case. The relatively high infection rate in the non-obliteration group may be overestimated due to small sample size. The re-intervention rate was 14.3% in the obliteration group and 17.4% in the non-obliteration group. These rates are in line with literature in which re-operation rates ranging from 10 to 12.1% are reported [9, 13, 16]. To prevent revision surgery in the future, we are now aware to always perform a meatoplasty when necessary.

There were no significant differences in ossicular chain status and in postoperative hearing between the two groups. With a median postoperative AC threshold level of 46.3 dB HL in the obliteration group and 42.5 dB HL in the non-obliteration group, the hearing results are to some degree disappointing. However, when interpreting these results one should take into account that the cohort consisted of very challenging chronically discharging ears of which most had several surgical interventions before. Therefore, the aim was to preserve hearing and not necessarily to improve hearing, which was achieved with a median change in AC threshold of 2.5 dB HL in the obliteration group and 0.0 dB HL in the non-obliteration group. Especially patients in the obliteration group should be able to wear hearing devices without any problems because of the restored anatomy. Unfortunately, it was not systematically reported in the patient records whether patients were able to.

### Limitations

Limitations of this study include the retrospective design and the possible indication bias. The mastoid obliteration with posterior canal wall reconstruction procedure was introduced in 2013 in our clinic and gradually replaced revision CWD without obliteration. Especially in the beginning of this transition, the choice to perform one of the two techniques was made by the surgeon based on preferences and experience. Later on, all surgeons adopted the obliteration technique and in principle no non-obliteration techniques were used anymore. Further limitations include the follow-up: the median follow-up duration was 20 months for the obliteration group and 67 months for the non-obliteration group. A shorter follow-up period may potentially overestimate the dry ear rate as long-term failures may not be included. However, no long-term failure was observed in the non-obliteration group and previous research has shown excellent long-term results after the obliteration technique [9, 13].

## Conclusion

We show that in our study population revision CWD surgery with mastoid obliteration and posterior canal wall reconstruction is superior to revision CWD surgery without mastoid obliteration in the management of chronically discharging mastoid cavities. In the obliteration group, a dry ear was achieved in 96.4% as this was 73.9% in the non-obliteration group. We found no differences in audiological outcome and in incidence of complications between the two techniques.

## Supplementary Information

Below is the link to the electronic supplementary material.

Supplementary file1 (DOCX 15 KB)
