# Supplementary material for: Revision surgery for chronically discharging mastoid cavities: mastoid obliteration with canal wall reconstruction versus non-obliteration surgery
Source: Eur Arch Otorhinolaryngol. 2021 Oct 27;279(8):3881–9. doi: 10.1007/s00405-021-07138-0 (PMC9249682; doi:10.1007/s00405-021-07138-0)
Supplement: Supplementary file 1 — Supplementary file1 (DOCX 15 KB) [file 405_2021_7138_MOESM1_ESM.docx]

**SUPPLEMENTAL DATA**

Supplemental table 1. Patient characteristics per surgical technique

|  | | **Obliteration with canal wall reconstruction** | **No obliteration** | **All patients** |
| --- | --- | --- | --- | --- |
| **Number of cases** | | 56 (70.9%) | 23 (29.1%) | 79 |
| **Median age in years (IQR)** | | 39.5 (28.0 – 53.8) | 41.0 (25.0 – 54.0) | 40.0 (28.0 – 54.0) |
| **Median follow-up in months (IQR)** | | 21.5 (15.5 – 43.8) | 67.0 (25.0 – 80.0) | 28.0 (17.0 – 55.0) |
| **Side** | **Left** | 27 (48.2%) | 14 (60.9%) | 41 (51.9%) |
|  | **Right** | 29 (51.8%) | 9 (39.1%) | 38 (48.1%) |
| **Sex** | **Male** | 30 (53.6%) | 13 (56.5%) | 43 (54.4%) |
|  | **Female** | 26 (46.4%) | 10 (43.5%) | 36 (45.6%) |
| **Pre-operative symptoms** | **Chronic discharge** | 55 (98.2%) | 23 (100%) | 78 (98.7%) |
|  | **Vertigo** | 10 (17.9%) | 0 (0%) | 11 (13.9%) |
|  | **Pain** | 5 (8.9%) | 1 (4.3%) | 6 (7.6%) |
|  | **Bothersome recurrent cleaning** | 1 (1.8%) | 0 (0%) | 1 (1.3%) |
| **Posterior canal wall reconstruction** | **Bone chips** | 40 (71.4%) | NA | 40 (50.6%) |
|  | **Bone chips with cartilage** | 9 (16.1%) | Na | 9 (11.4%) |
|  | **Cartilage** | 7 (12.5%) | NA | 7 (8.9%) |
|  | **No reconstruction** | NA | 23 (100%) | 23 (29.1%) |
| **Obliteration material** | **Autologous bone dust** | 45 (80.4%) | 19 (82.6%)* | 64 (81.0%) |
|  | **Autologous bone dust with bioactive glass granules** | 9 (16.1%) | 0 (0%) | 9 (11.4%) |
|  | **Bioactive glass granules** | 2 (3.6%) | 0 (0%) | 2 (2.5%) |
| **Meatoplasty** | **Yes** | 17 (30.4%) | 10 (43.5%) | 27 (34.2%) |
|  | **No** | 39 (69.6%) | 13 (56.5%) | 52 (65.8%) |

IQR, inter quartile range; NA, not applicable

* in 19 cases (82.6%) in the no-obliteration group the mastoid was smoothened with some bone dust.

Supplemental table 2. Ossicular chain status and ossicular chain reconstruction per surgical technique.

|  | | **Obliteration with canal wall reconstruction** | **No obliteration** | **P** |
| --- | --- | --- | --- | --- |
| **Number of cases** | | 56 (70.9%) | 23 (29.1%) |  |
| **Perioperative ossicular chain status** | **Intact chain** | 6 (10.7%) | 3 (13%) | .767 |
|  | **Incus absent** | 22 (39.3%) | 12 (52.2%) | .293 |
|  | **Stapes superstructure absent** | 1 (1.8%) | 0 | NA |
|  | **Incus and stapes superstructure absent** | 22 (39.3%) | 4 (17.4%) | .060 |
|  | **No ossicular chain remnants** | 0 | 1 (4.3%) | NA |
|  | **Unknown** | 5 (8.9%) | 3 (13%) | .582 |
| **Ossicular chain reconstruction** | **No reconstruction** | 30 (53.6%) | 11 (47.8%) | .642 |
|  | **Intact chain** | 1 (1.8%) | 0 | NA |
|  | **Incus interposition** | 3 (5.4%) | 0 | NA |
|  | **PORP** | 3 (5.4%) | 2 (8.7%) | .580 |
|  | **TORP** | 11 (19.6%) | 1 (4.3%) | .081 |
|  | **TM directly on footplate** | 1 (1.8%) | 1 (4.3%) | .510 |
|  | **TM on stapes (type III reconstruction)** | 7 (12.5%) | 8 (34.8%) | **.022** |

PORP, partial ossicular replacement prosthesis; TORP, total ossicular replacement prosthesis; TM, tympanic membrane.
